# Supplementary material for: Graded phononic metamaterials based on scalable microfabrication and design
Source: Nat Commun. 2026 Feb 25;17:3192. doi: 10.1038/s41467-026-69888-x (PMC13057018; doi:10.1038/s41467-026-69888-x)
Supplement: Supplementary file 1 — Supplementary Information [file 41467_2026_69888_MOESM1_ESM.pdf]

# Supplementary Information for: Graded phononic metamaterials based on scalable microfabrication and design

Charles Dorn[†1,2], Vignesh Kannan[†1,3], Ute Drechsler[4], and Dennis M. Kochmann[*1]

[1]Mechanics and Materials Laboratory, ETH Zurich, 8092 Zurich, Switzerland
[2]Department of Aeronautics and Astronautics, University of Washington, Seattle, WA 98195, USA
[3]Laboratoire de Mécanique des Solides, CNRS, École Polytechnique, Institut Polytechnique de Paris, 91128 Palaiseau, France
[4]IBM Research – Zurich, 8803 Rüschlikon, Switzerland

## Supplementary Note 1 Inverse design

We provide a detailed description of the inverse design methodology used to generate the designs presented in Figs. 1 and 2 of the main text. Our procedure for the inverse design is composed of the following three steps. First, a unit cell design space is defined and dispersion relations are computed throughout the design space, which is discussed in Supplementary Note 1.1. Second, tiles with spatially graded arrangements of unit cells are designed by optimizing ray trajectories, for which the mathematical foundation and numerical implementation are discussed in Supplementary Note 1.2 and Supplementary Note 1.3, respectively. Finally, the third step is to assemble the tiles into a desired wave guiding configuration, which is discussed in Supplementary Note 1.4.

### Supplementary Note 1.1 Defining a design space

The first step is to define a parameterized design space of unit cells, which determines the variables that can be spatially graded in a metamaterial consisting of many unit cells. In this work, we define the unit cell design space shown in Fig. 1a of the main text, consisting of elastic beams along each edge, square lattice vectors, and a single geometric design variable $\theta$. This is one of many possible choices for a unit cell design space, but it was carefully selected for the following reasons:

- We consider beam-based unit cells, for which beam finite elements provide an efficient and physically realistic model (for a comparison of beam elements, solid elements, and experiments in the context of dispersion relations see [1]). Furthermore, planar beam architectures exhibit decoupling between in- and out-of-plane modes for the lowest dispersion surfaces [1]. Since it is experimentally feasible to excite and measure out-of-plane motion in the wafer samples presented here, this decoupling is advantageous.

[†]These authors contributed equally to this work.
[*]Corresponding author: dmk@ethz.ch

- For computational efficiency, we define the unit cell design space to have a single parameter (though the approach is sufficiently general to extend to arbitrarily many design parameters). Since the dispersion relation and its first and second derivatives must be numerically evaluated, defining a single parameter design space maximizes efficiency for optimizing complex spatial gradings.
- A well-chosen unit cell design parameter must be such that its variation leads to a significant change in the dispersion relations. This is essential for enabling wave guiding in graded architectures — and is the case for $\theta$ in our study. For example, the dispersion surfaces in Fig. 1a of the main text differ by a factor of nearly 4 in their peak value at the extremes of the design space. This allows for substantially different local dispersion relations in different spatial locations in a graded metamaterial. In contrast, if the dispersion surface hardly changes throughout the design space, then the spatial grading would have little effect on how waves propagate and limit the functionality of grading.
- The unit cell should have four-fold symmetry. This enables the tiles (e.g., Fig. 1b of the main text) to be rotated by 90° and mirrored when placed into tile assemblies (e.g., Fig. 2a and d of the main text) without introducing sharp interfaces in unit cell geometry between adjacent tiles.

Considering these four factors, the choice of the unit cell design space in Fig. 1c of the main text is justified, though other architectures can also be considered within our design framework. We also note that, throughout this paper, we consider the lowest out-of-plane dispersion surface only to simplify modeling and experiments, but the presented methodology can readily be applied to higher dispersion branches since ray theory is valid at high frequencies [2, 3].

Throughout the simulations, physical units are used that match the experimental configuration. The unit cell height and width is $L = 100\,\mu$m, the beam thickness is $10\,\mu$m, and the beam width is $5\,\mu$m. The lowest dispersion surfaces computed using beam finite elements at the two extremes of the design space are plotted on the first Brillouin zone in Fig. 1c of the main text; details of the finite element (FE) model used to compute the dispersion relations are discussed in Supplementary Note 2.1.

## Supplementary Note 1.2 Theoretical framework for inverse design

The tile design step involves optimization of a ray tracing model of wave propagation. Here, ray tracing is used for forward modeling of how waves propagate through spatially graded metamaterials. We refer to [4] for a detailed derivation and description of ray tracing in graded metamaterials. We build an inverse design framework around ray tracing by extending the formulation of [2] to handle more general cost functions suitable for tile design.

The starting point is forward modeling of wave propagation in a planar metamaterial using ray tracing. Given the *local* dispersion relation $\omega(\boldsymbol{k}, \boldsymbol{x})$, which relates the wave vector $\boldsymbol{k} \in \mathbb{R}^2$ to the frequency $\omega \in \mathbb{R}$, at a given position $\boldsymbol{x} \in \mathbb{R}^2$ (e.g., computed by Bloch wave analysis for the unit cell at position $\boldsymbol{x}$), wave propagation is described by the ray trajectories satisfying

$$\dot{\boldsymbol{x}} = \frac{\partial \omega}{\partial \boldsymbol{k}}, \tag{S1}$$

$$\dot{\boldsymbol{k}} = -\frac{\partial \omega}{\partial \boldsymbol{x}} \tag{S2}$$

This constitutes the *ray tracing system*: a system of first-order differential equations that is solved, given initial conditions $\boldsymbol{x}_0 = \boldsymbol{x}(t = 0)$ and $\boldsymbol{k}_0 = \boldsymbol{k}(t = 0)$. Eq. (S1) states that the the group velocity $\boldsymbol{V} = \frac{\partial \omega}{\partial \boldsymbol{k}}$ is tangent to ray path $\boldsymbol{x}(t)$. Eq. (S2) states that changes in wave vector $\boldsymbol{k}$ along the ray path are driven by the spatial change in the local dispersion relation. The ray tracing system can be solved for many rays to efficiently capture where a wave propagates in a graded metamaterial.

Ray tracing provides an efficient forward modeling tool that is particularly advantageous for inverse problems, since ray parameters are differentiable. However, ray theory is an approximation based on the assumption of

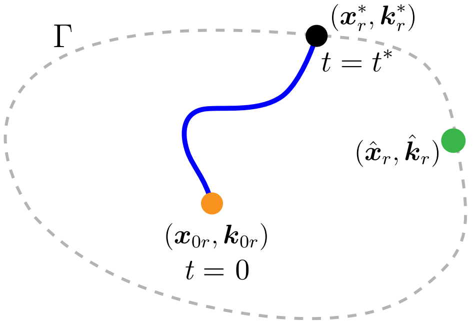


**Supplementary Figure 1: Definitions for setting up the cost function.** Ray $r$ has initial conditions $(\boldsymbol{x}_{0r}, \boldsymbol{k}_{0r})$ and passes through the exit curve $\Gamma$ at time $t^*$ with state $(\boldsymbol{x}_r^*, \boldsymbol{k}_r^*)$. The optimization seeks to align the exit state of the ray with the desired exit state $(\hat{\boldsymbol{x}}_r, \hat{\boldsymbol{k}}_r)$.

a separation of scales between the wavelength (on a short length scale) and the spatial variation of unit cells (on a long length scale). For sufficiently aggressive gradings with respect to wavelengths of interest, the ray solution will introduce error and fail to capture phenomena such as mode coupling. It is an open problem to provide rigorous, quantitative validity conditions for ray theory in dispersive, microstructured media; only in simpler settings such as isotropic continuum solids have validity conditions have been studied, e.g., in [5, 6]. Thus, in our approach, we rely on ray tracing solutions to enable inverse design (due to their efficiency and differentiability), followed by numerical validation (via transient dynamic FE simulations) and experimental validation to confirm that the ray solution is representative.

Leveraging ray tracing as an efficient modeling tool, we formulate an optimization problem to design the spatial distribution of unit cells to shape the ray trajectories. Specifically, consider a ray $r$ emerging from an initial position $\boldsymbol{x}_{0r}$ with initial wave vector $\boldsymbol{k}_{0r}$. A contour $\Gamma$ is drawn to enclose $\boldsymbol{x}_{0r}$ that we define as the *exit curve*, which is fixed (see Supplementary Figure 1). Denote $\boldsymbol{x}_r^*$ and $\boldsymbol{k}_r^*$, respectively, as the position and wave vector at which ray $r$ first crosses the exit curve, which occurs at time $t_r^*$. We aim to force the ray to cross through the exit curve at a specified target point $\hat{\boldsymbol{x}}_r$ with a target wave vector $\hat{\boldsymbol{k}}_r$. To achieve the prescribed exit conditions, we minimize the cost function

$$c_r(\boldsymbol{x}_r^*(\boldsymbol{\theta}), \boldsymbol{k}_r^*(\boldsymbol{\theta})) = w_1 ||\boldsymbol{x}_r^*(\boldsymbol{\theta}) - \hat{\boldsymbol{x}}_r||^2 + w_2 ||\boldsymbol{k}_r^*(\boldsymbol{\theta}) - \hat{\boldsymbol{k}}_r||^2, \tag{S3}$$

where $\boldsymbol{\theta} \in \mathbb{R}^N$ is a vector capturing the spatial distribution of design variables, i.e., $\boldsymbol{\theta}$ is the vector collecting the design variables at all points within the design domain (see Supplementary Note 1.3 for details on defining $\boldsymbol{\theta}$). Constants $w_1, w_2 \geq 0$ weight each cost function term. Supplementary Figure 1 schematically illustrates the variables in the cost function. We define a constrained optimization problem to minimize $c_r$ of $n_r$ rays, which takes the form

$$\begin{aligned} \min_{\boldsymbol{\theta}} C(\boldsymbol{\theta}) &= \sum_{r=1}^{n_r} c_r(\boldsymbol{x}_r^*(\boldsymbol{\theta}), \boldsymbol{k}_r^*(\boldsymbol{\theta})) \\ \text{subject to} \quad \dot{\boldsymbol{x}}_r &= \frac{\partial \omega}{\partial \boldsymbol{k}}, \qquad t \in [0, t_r^*], \quad r = 1, ..., n_r, \\ \dot{\boldsymbol{k}}_r &= -\frac{\partial \omega}{\partial \boldsymbol{x}}, \qquad t \in [0, t_r^*], \quad r = 1, ..., n_r, \\ \mathcal{G}_r(\boldsymbol{x}_r^*) &= 0, \qquad r = 1, ..., n_r. \end{aligned} \tag{S4}$$

Here, the ray tracing system of Eqs. (S1) and (S2) is treated as constraints. Since the exit time $t_r^*$ is not fixed, we impose the additional constraint $\mathcal{G}_r(\boldsymbol{x}_r^*) = 0$. The quantity $\mathcal{G}_r$ is defined as the signed distance of the ray at time $t_r^*$ to the exit curve $\Gamma$, so that $\mathcal{G}_r(\boldsymbol{x}_r^*) = 0$ enforces $t_r^*$ to be the time at which the ray exits $\Gamma$ (see [2] and [7] for further discussion).

The optimization problem of Eq. (S4) is solved using gradient-based optimization, for which the total derivative of the cost function with respect to the design variables, i.e., $\frac{\mathrm{d}C}{\mathrm{d}\boldsymbol{\theta}}$ is required. To efficiently compute this

quantity, the adjoint state method is used. Consider the Lagrangian corresponding to the cost function of a single ray (with the subscript $r$ omitted for clarity),

$$\mathcal{L}(\boldsymbol{\theta}, \boldsymbol{x}, \boldsymbol{k}, \boldsymbol{\lambda}, \boldsymbol{\mu}, \rho, t^*) = C - \int_0^{t^*} \left[ \boldsymbol{\lambda}^{\intercal} \left( \dot{\boldsymbol{x}} - \frac{\partial \omega}{\partial \boldsymbol{k}} \right) + \boldsymbol{\mu}^{\intercal} \left( \dot{\boldsymbol{k}} + \frac{\partial \omega}{\partial \boldsymbol{x}} \right) \right] \mathrm{d}t - \rho \mathcal{G}, \tag{S5}$$

where $\boldsymbol{\lambda}$, $\boldsymbol{\mu}$, and $\rho$ are the Lagrange multipliers corresponding to the three constraint sets of Eq. (S4), respectively.

The adjoint state method proceeds by enforcing stationarity of the Lagrangian with respect to all variables except $\boldsymbol{\theta}$. Setting the variations with respect to the Lagrange multipliers $\boldsymbol{\lambda}$, $\boldsymbol{\mu}$, and $\rho$ to zero directly return the constraints of Eq. (S4). Setting variations with respect to the state variables $\boldsymbol{x}$ and $\boldsymbol{k}$ to zero returns the adjoint system, which takes the form

$$\dot{\boldsymbol{\lambda}} = -\frac{\partial^2 \omega}{\partial \boldsymbol{x} \partial \boldsymbol{k}} \boldsymbol{\lambda} + \frac{\partial^2 \omega}{\partial \boldsymbol{x} \partial \boldsymbol{x}} \boldsymbol{\mu}, \tag{S6}$$

$$\dot{\boldsymbol{\mu}} = -\frac{\partial^2 \omega}{\partial \boldsymbol{k} \partial \boldsymbol{k}} \boldsymbol{\lambda} + \frac{\partial^2 \omega}{\partial \boldsymbol{k} \partial \boldsymbol{x}} \boldsymbol{\mu}, \tag{S7}$$

with end conditions

$$\boldsymbol{\lambda}(t^*) = \frac{\partial C}{\partial \boldsymbol{x}^*} - \rho \frac{\partial \mathcal{G}}{\partial \boldsymbol{x}^*}, \tag{S8}$$

$$\boldsymbol{\mu}(t^*) = \frac{\partial C}{\partial \boldsymbol{k}^*}. \tag{S9}$$

Eqs. (S6), (S7), and (S8) are identical to the adjoint system in [2]. Here, the end condition for $\boldsymbol{\mu}$ in Eq. (S9) is nonzero, because we consider cost functions dependent on $\boldsymbol{k}$ (which is not the case in [2]). This is essential in our tile design, as it allows us to control the wave vector with which rays enter and/or leave a tile.

An equation for $\rho$ is obtained by enforcing stationarity of the Lagrangian with respect to $t^*$,

$$\rho = \frac{\frac{\partial C}{\partial \boldsymbol{x}^*} \cdot \boldsymbol{v}^* - \frac{\partial C}{\partial \boldsymbol{k}^*} \cdot \frac{\partial \omega}{\partial \boldsymbol{x}^*}}{\frac{\partial \mathcal{G}}{\partial \boldsymbol{x}^*} \cdot \boldsymbol{v}^*}. \tag{S10}$$

The form of $\rho$ in Eq. (S10) differs from that in [2], again because here we consider a cost function dependent on $\boldsymbol{k}^*$.

Finally, since variations of the Lagrangian with respect to all variables except $\boldsymbol{\theta}$ are zero, the gradient of the cost function is easily obtained from

$$\frac{\mathrm{d}C}{\mathrm{d}\boldsymbol{\theta}} = \frac{\delta \mathcal{L}}{\delta \boldsymbol{\theta}} = -\int_0^{t^*} \left( -\boldsymbol{\lambda}^{\intercal} \frac{\partial^2 \omega}{\partial \boldsymbol{k} \partial \boldsymbol{\theta}} + \boldsymbol{\mu}^{\intercal} \frac{\partial^2 \omega}{\partial \boldsymbol{x} \partial \boldsymbol{\theta}} \right) \mathrm{d}t, \tag{S11}$$

where all quantities in the integrand are known after forward and reverse ray tracing.

The formulations of this section generally follow those of [2], which provides more detailed derivations and explanations. However, we here consider two distinct differences to generalize the framework and make it suitable for tile design. First, an arbitrary exit curve is considered here in contrast to a strictly circular exit curve in [2]. Second, the cost function defined in Eq. (S3) is a function of both $\boldsymbol{x}^*$ and $\boldsymbol{k}^*$, while [2] considers cost functions only dependent on $\boldsymbol{x}^*$. Consequently, Eqs. (S9) and (S10) differ from the analogous equations (19) and (12) of [2] due to contributions from $\boldsymbol{k}^*$-dependence of the cost function $C$.

## Supplementary Note 1.3 Numerical implementation of tile design

Using the optimization framework of Supplementary Note 1.2, we present the design of two tiles, which are shown in Fig. 1d of the main text. In this case, the optimization aims to design the spatial distribution

of the single unit cell design variable $\theta$ to satisfy two different objective functions that are outlined in Sections Supplementary Note 1.3.1 and Supplementary Note 1.3.2.

Since ray tracing solutions conserve frequency (as long as the local dispersion relation is time-independent, which is the case for linear elastic materials), each ray has a fixed frequency. We consider optimization of many rays at the same fixed frequency $\omega_0$, which we take as $\omega_0 = 750$ kHz throughout all examples. One could consider rays of multiple frequencies in the objective function, as was done in [2], but we observed that our designs exhibited sufficiently broad frequency range surrounding the design frequency to demonstrate experimentally. Supplementary Note 2.2 discusses further the behavior at different frequencies.

For both tiles, a vector $\boldsymbol{\theta} \in \mathbb{R}^N$ is defined to parameterize the design problem. Entries of $\boldsymbol{\theta}$ correspond to values of $\theta$ on a regular $n \times n$ square grid, such that $N = n^2$. We consider a design grid that spans $64\times64$ unit cells, corresponding to the size of the tile. The spacing of the grid of design variables may or may not align with the physical lattice of unit cells. This allows for control over the overall number of design variables. If the design variable grid is taken to have the same spacing as the unit cells, then each design variable corresponds to one unit cell's design. Alternatively, a coarser grid of design variables can be chosen, from which the design of a specific unit cell can be interpolated. Control over the coarseness of the design grid allows for adaptive spatial refinement during optimization, which is beneficial for finding spatially smooth optimal solutions [2, 7]. We begin the optimization with a design grid spacing of $16L$ and gradually refine it to a spacing of $L$ (such that each unit cell has its own entry in $\boldsymbol{\theta}$) during the optimization.

Following the formulations of Supplementary Note 1.2, a three-step procedure is followed to compute the cost function gradient. First, given a set of ray initial conditions and design, the ray is traced by solving Eqs. (S1)-(S2). Second, the adjoint system of Eqs. (S6)-(S7) is solved, starting from the end of the ray and marching backwards in time. Finally, the cost function gradient is computed by evaluating the integral of Eq. (S11) along each ray, which is given to an optimization algorithm to update the design variables. A fourth-order Runge-Kutta solver is used to solve both the ray tracing and adjoint systems, while trapezoidal integration along the ray path is used to evaluate Eq. (S11). The computed cost function gradient is used to inform the L-BFGS algorithm [8] for gradient-based optimization, for which we use the NLopt implementation [9] for all examples.

A key challenge in this optimization is that we seek solutions that are not only global minima, but which send the cost function of Eq. (S4) to zero such that the desired exit conditions are satisfied for each ray. This is critical for tile assembly, since any misalignment in the ray trajectories at the exit of a tile results in a misaligned incident wave to the adjacent tile, thus corrupting the wave guiding capabilities in assemblies of tiles. In the presented examples, there was sufficient design freedom and smoothness to find solutions that zero the cost function, using gradient-based optimization. While we demonstrate two examples of square tiles, our optimization framework offers a flexible cost function definition to pursue solutions where rays pass through the exit curve with an arbitrary prescribed position and direction. While we exemplify cases where rays exit perpendicular to the tile edge to enable modular assembly of tiles, future work could explore alternative objectives, as well as global optimization, in pursuit of tile designs with additional functionalities.

### Supplementary Note 1.3.1 Tile 1: Point source to plane wave

The objective of Tile 1 is to convert a point excitation at its center to plane waves exiting the four tile edges (i.e., rays exiting perpendicular to the tile edges). For this example, we take $n_r = 100$ rays in in the cost function. The initial conditions of each ray are determined by the point excitation at the center, such that $\boldsymbol{x}_{0r} = \mathbf{0}$ for all rays. The set of all possible wave vector initial conditions corresponding to the excitation frequency lie on the isofrequency contour of the local dispersion surface at the excitation point, i.e., $\omega(\boldsymbol{k}, \theta_0) = \omega_0$ where $\theta_0 = \theta(\boldsymbol{x}_0)$. The direction that the ray exits the excitation point is the direction of the vector $\boldsymbol{V}(\boldsymbol{k}_{0r}, \boldsymbol{x}_{0r})$ (i.e., the direction of the initial group velocity). We choose values of $\boldsymbol{k}_{0r}$ such that the rays uniformly span initial angles from 0 to 45° and enforce eight-fold symmetry in the distribution of $\theta$ over the tile, such that the rays between 0 and 45° can be rotated/reflected to capture all rays exiting the point source.

The unit cell design parameter $\theta$ is set to a fixed value $\theta_0$ at the point $\boldsymbol{x}_0$ to ensure that the local dispersion relation at the excitation point remains constant throughout the optimization, so that each ray's initial conditions remain constant as well. Additionally, $\theta$ is fixed at $\theta_0$ on the boundary of the tiles (at $x = \pm 32L$ and $y = \pm 32L$) as well as everywhere outside of the tile, which ensures continuity in $\theta$ between adjacent tiles during the tile assembly step. In this study, we found through numerical experiments that $\theta_0 = 0.4$ is a suitable value.

To achieve the design of Tile 1, the exit contour is defined as a semicircle to the left of $x = 64L$ with a radius of $64L$. In the cost function of Eq. (S3), only the exit conditions $\hat{x} = 0$ and $\hat{k}_y = 0$ are included. The $y$-coordinate of the exit point is left free, and $k_x$ at the exit does not need to be prescribed due to the four-fold symmetry of the dispersion relations (enforcing $k_y = 0$ is sufficient for ensuring the group velocity is horizontal). The corresponding cost function seeks rays that exit the plane at $x = 32L$ horizontally. Finally, eight-fold symmetry in the $\theta$-distribution is enforced about $x = 0$, $y = 0$, $x = y$, and $x = -y$. These symmetries are implemented by mirroring the cost function gradient about these planes to directly enforce symmetry before handing it to the optimizer). The resulting ray trajectories can be mirrored about the symmetry axes, as plotted in Fig. 1d of the main text.

The ray solution allows for characterization of wave propagation properties through the tile. In Tile 1, the maximum and minimum wavelengths among all rays are 333.0 $\mu$m (or $3.33L$) and 254.3 $\mu$m (or $2.543L$), respectively. Additionally, the maximum and minimum group velocity magnitudes among all rays are 480.0 and 342.0 m/s, respectively.

### Supplementary Note 1.3.2 Tile 2: Plane wave $90°$ turn

The objective of Tile 2 is to steer a plane wave incident to the left boundary of the tile to a plane wave exiting out the bottom of the tile. We consider $n_r = 80$ rays in the cost function. Around the perimeter of the tile, the unit cell design is fixed at $\theta_0 = 0.4L$ to ensure compatibility with other tiles. Rays are considered with an initial $x_0 = -32L$ and uniformly spaced $y$-coordinates spanning $y = \pm 20L$ (for a $64L \times 64L$ tile centered at the origin). The initial wave vector is the same for all rays, which is obtained by taking the point on the isofrequency contour $\omega(\boldsymbol{k}, \theta_0) = \omega_0$ corresponding to a group velocity $\boldsymbol{V}(\boldsymbol{k}_0, \theta_0)$ that is horizontal.

In the cost function, the target exit wave vector is $\hat{k}_x = 0$ with $\hat{k}_y$ left unspecified, which is sufficient for enforcing that the rays exit vertically due to the four-fold symmetry of the dispersion relations. To ensure the rays exit out the bottom of the tile, we take $\hat{y} = -32L$. The value of $\hat{x}$ is defined independently for each ray as $\hat{x} = y_0$, which was carefully chosen to allow a solution mimicking an interface that deflects rays downward, as observed in the plot of Fig. 1d in the main text. We found this to be a reliable approach to finding solutions for turning a plane wave by $90°$. In the resulting design, the interface is diffusely spread over several unit cells due to the spatial grading, so that the ray trajectories remain smooth and a sharp interface is avoided. The exit curve is taken as a semicircle of radius $64L$ centered at the bottom left corner of the tile. We enforce symmetry of the $\theta$-distribution about $x = y$, which we found to aid in converging to a solution.

In Tile 2, the maximum and minimum wavelengths among all rays are 333.4 $\mu$m (or $3.334L$) and 221.4 $\mu$m (or $2.214L$), respectively. Additionally, the maximum and minimum group velocity magnitudes among all rays are 669.8 and 259.9 m/s, respectively.

## Supplementary Note 1.4 Tile assembly

Since each tile is designed to be compatible with an equal and constant value of $\theta = 0.4L$ around all boundaries, their rays can be connected to construct a continuous solution when the two tiles are placed next to each other. This is because the ray tracing equations depend only on the local dispersion relations, which we have ensured are continuous across tile boundaries. Thus, taking these tiles as building blocks can lead to many customized tile assemblies that guide waves in different ways. We note that since the dispersion

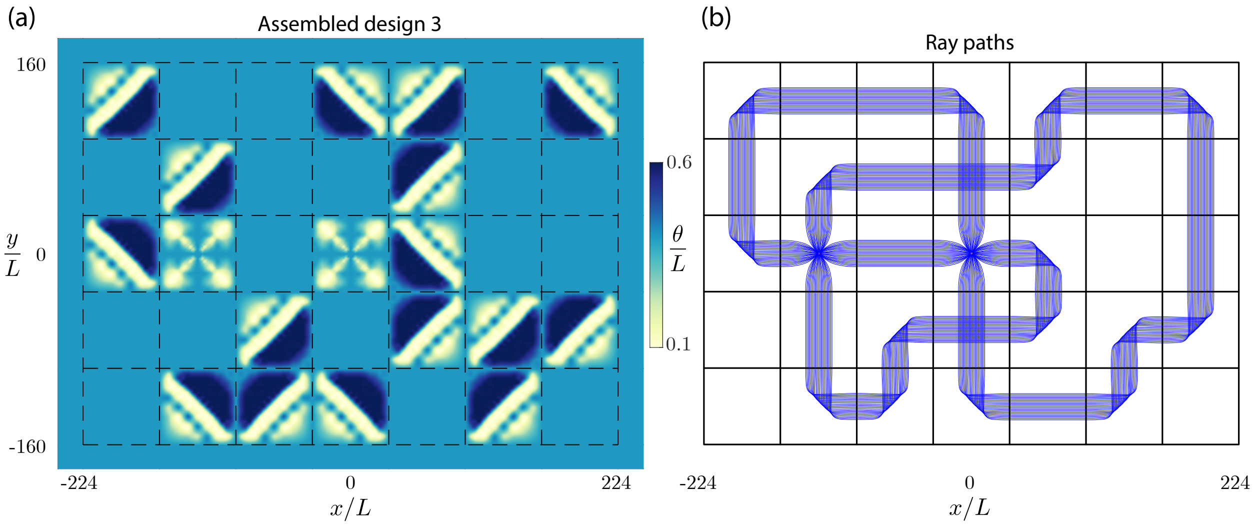


**Supplementary Figure 2: Demonstration of a complex tile assembly.** An additional waveguide design based on Tiles 1 and 2 spans approximately 143,000 unit cells. (a) Spatial distribution of the unit cell design variable $\theta$, with dotted lines marking tile boundaries. (b) The corresponding ray trajectories.

relations exhibit four-fold symmetry about the $k_x = 0$ and $k_y = 0$ axes (following the four-fold symmetry of the unit cell geometry), the tiles can be reflected and rotated during their assembly while ensuring their ray solutions follow the same reflections/rotations. Two examples of tile assemblies are presented in Fig. 2a and 2d of the main text. Both designs are validated by transient finite element simulations (see Section Supplementary Note 2), and the design of Fig. 2a is also demonstrated experimentally. An additional assembled design example is shown in Supplementary Fig. 2, which demonstrates scalability of the modular tile-based design approach to achieve a more complex guided wave path. This design spans approximately 143,000 unit cells to guide waves emerging from either instance of Tile 1 along a complicated path corresponding to the rays of Supplementary Fig. 2b.

# Supplementary Note 2 Finite element modeling

We use an FE model of beam-based metamaterials to perform two types of computations. The first is the computation of dispersion relations for unit cells throughout the design space, which is the initial step in the numerical inverse design process. The second use is transient dynamic simulations on a finite domain containing the assembled design to validate its wave guiding capability and enable quantitative comparison to experiment.

For both computations, Timoshenko beam finite elements are used to discretize the metamaterial, which is a reliable and efficient approach for physically realistic modeling of wave propagation in beam lattices [1]. The open-source C++ finite element code *ae108* [10] is used. We use meshes with a maximum element size of $L/10$, which was found to be sufficiently dense to accurately capture the lowest out-of-plane mode. The orthotropic anisotropy of silicon is accounted for by using the directional moduli determined by the in-plane orientation of each beam. In the prototype and the FE model, the lattice vectors are aligned with the $\bar{1}10$ and 110 directions of the silicon crystal structure; see [11] for the wafer orientation definitions and corresponding orthotropic moduli, which were adopted here.

## Supplementary Note 2.1 Dispersion relation computations

Dispersion surfaces are computed based on a finite element model of a unit cell. For a given unit cell, the computational procedure involves first applying Bloch boundary conditions to the unit cell and solving the resulting eigenvalue(s) for a given wave vector $\boldsymbol{k}$; see, e.g., [12]. Dispersion surfaces are constructed by solving the Bloch eigenvalue problem for values of $\boldsymbol{k}$ spanning the first Brillouin zone. For planar unit cells modeled with beam elements, in- and out-of-planes are decoupled [13]. That is, out-of-plane excitation will only excite out-of-plane motion. Since we experimentally measure out-of-plane motion, we limit our interest to dispersion surfaces corresponding to out-of-plane mode shapes. Without loss of generality, we only consider the lowest out-of-plane dispersion surface (agreement between simulation results and our experiments confirm that this is indeed sufficient).

The inverse design framework requires the dispersion relation $\omega(k_1, k_2, \theta)$ and its first and second partial derivatives to be evaluated for a given wave vector and $\theta$-value. To obtain the dispersion surface throughout the unit cell design space ($0.1L \leq \theta \leq 0.6L$), we solve the Bloch eigenvalue problem on a $50 \times 50$ grid in $k$-space, spanning the first Brillouin zone for 100 values of $\theta$ ranging from $0.1L$ to $0.6L$. The dispersion surfaces corresponding to $\theta = 0.1L$ and $\theta = 0.6L$ are shown in Fig. 1a of the main text. Finite differences are used to approximate the first and second derivatives of the dispersion surface with respect to $\boldsymbol{k}$ and $\theta$, which are also needed for optimization. Evaluations of the dispersion surface and its derivatives for any given values of $\boldsymbol{k}$ and $\theta$ are then obtained by interpolation.

## Supplementary Note 2.2 Transient dynamic simulations

Transient finite element simulations are performed to numerically validate the designed tile assemblies of Fig. 2 of the main text, as well as for comparison to experiments. For simulations of both designs, a finite domain of 300×300 unit cells (3 cm × 3 cm) is modeled. To match the experimental setting, a pulse displacement excitation is applied in the out-of-plane direction at the junction of the unit cell at the center of the center tile (at $x = y = 0$). The pulse is a half-sine wave of duration 1 $\mu s$. While not exactly representative of the broadband experimental pulse excitation, this idealized excitation spans approximately the same frequency range. The outer boundary of the simulation domain is clamped and the entire domain is initially at rest. A Newmark-beta integration scheme (with parameters $\gamma = 0.5$ and $\beta = 0.25$, thus avoiding numerical damping) is used to solve for the dynamic response with a time step of $0.22\,\mu$s.

For the figure-eight design of Fig. 2a of the main text, the response is simulated for a duration of 55 $\mu$s, which is sufficient time for the wave to complete the figure-eight trajectory. Snapshots of the resulting wave field are plotted in Supplementary Fig. 3a-d at times $t = 13$, 26, 39, and 52 $\mu$s, where the color map corresponds to the instantaneous out-of-plane displacement. The simulation results show strong wave guiding despite the broadband excitation. Note that, as no dissipative boundary conditions are imposed (all boundaries are clamped) and the dynamic solver suppresses numerical damping, any observed wave attenuation and redirection effects stem from the spatially graded design.

Although the spatial grading is designed specifically for wave guiding at 750 kHz, the operating bandwidth spans a much wider range than the immediate vicinity of the design frequency. This is evident in Supplementary Fig. 3e-h, which shows the amplitude of the frequency spectrum of the out-of-plane displacement, denoted $\tilde{u}_z(\boldsymbol{x}, f)$, at frequency snapshots $f = 250$, 500, 750, and 1000 kHz. While the cleanest wave guiding is observed at the design frequency of 750 kHz, strong wave guiding is observed down to 250 kHz.

For the cross design of Fig. 2d of the main text, the response is simulated for a duration of $104\,\mu$s to allow sufficient time for the wave to travel along the entire design trajectory. Snapshots of the resulting wave field are plotted in Supplementary Fig. 4a-d at times $t = 13$, 52, 78, and 104 $\mu$s, where the color map corresponds to the instantaneous out-of-plane displacement. Again, wave guiding is observed over a broad frequency spectrum, as shown in the snapshots of the frequency spectrum in Supplementary Fig. 4.

For clear visualization of wave guiding, we also performed transient simulations with harmonic excitation at

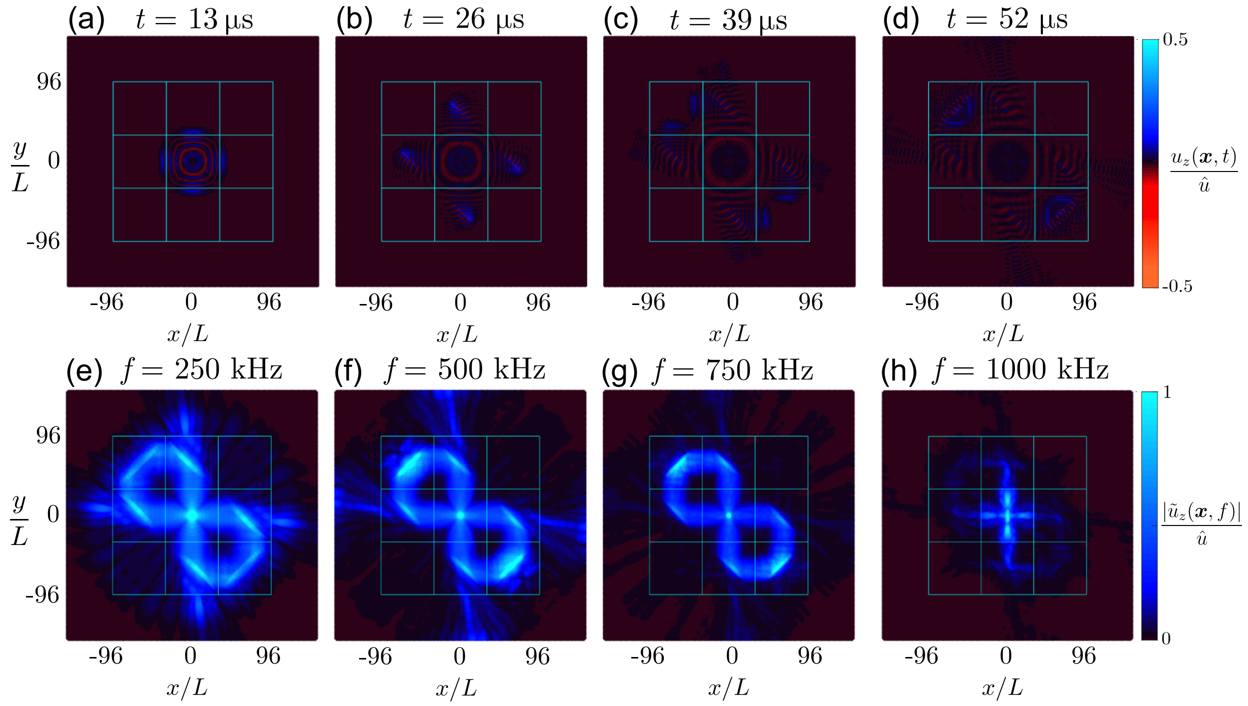


**Supplementary Figure 3: Broadband transient simulation results for the figure-eight design.** Snapshots of the wave field in the transient dynamic finite element simulation of the figure-eight design at (a) 13 $\mu$s, (b) 26 $\mu$s, (c) 39 $\mu$s, and (d) 52 $\mu$s. The amplitude of the temporal FFT of the displacement signal at each spatial location is plotted at (e) 250 kHz, (f) 500 kHz, (g) 750 kHz (the design frequency), and (h) 1000 kHz.

the design frequency $f = 750$ kHz. The results are shown in Supplementary Videos 1 and 2.

## Supplementary Note 2.3 Computation time

Efficient forward modeling based on ray tracing is key for enabling the inverse design in our study. There is a stark contrast in computation time between ray tracing and transient dynamic FE simulations. To compare these solution methods, we consider the example of the figure-eight design. In the transient dynamic FE simulation, following the setup described in Supplementary Note 2.2, the resulting model has 2,170,946 elements and 1,990,945 nodes, following a convergence study confirming this is a sufficiently refined mesh for the frequencies considered. The simulation was implemented in ae108 [10], an in-house parallelized C++ finite element code. For a dynamic simulation on this mesh over 248 time steps, the overall computation time was 5.02 hours, when run in parallel on 16 cores.

The ray tracing solution provides a significantly more efficient means of evaluating the forward problem. This efficiency stems from solving small systems of first-order ordinary differential equations instead of solving the full elastodynamic partial differential equation. Additionally, the solution of each ray may be evaluated independently, offering control to only evaluate the solution in a region of interest as well as the opportunity for parallelization. While the exact computation time of tracing a ray may vary, to provide an idea of the computational effort, the average time to trace one ray through Tile 1 (among the 100 rays considered) is 0.41 seconds in our Matlab implementation. The 100 rays considered to capture the Tile 1 solution can be evaluated in 5.15 seconds when running on 14 cores. We note here that the ray tracing system is defined using precomputed dispersion relations over the design space of unit cells; precomputation of dispersion relations is not included in the listed computation times, as this step needs to be performed offline once.

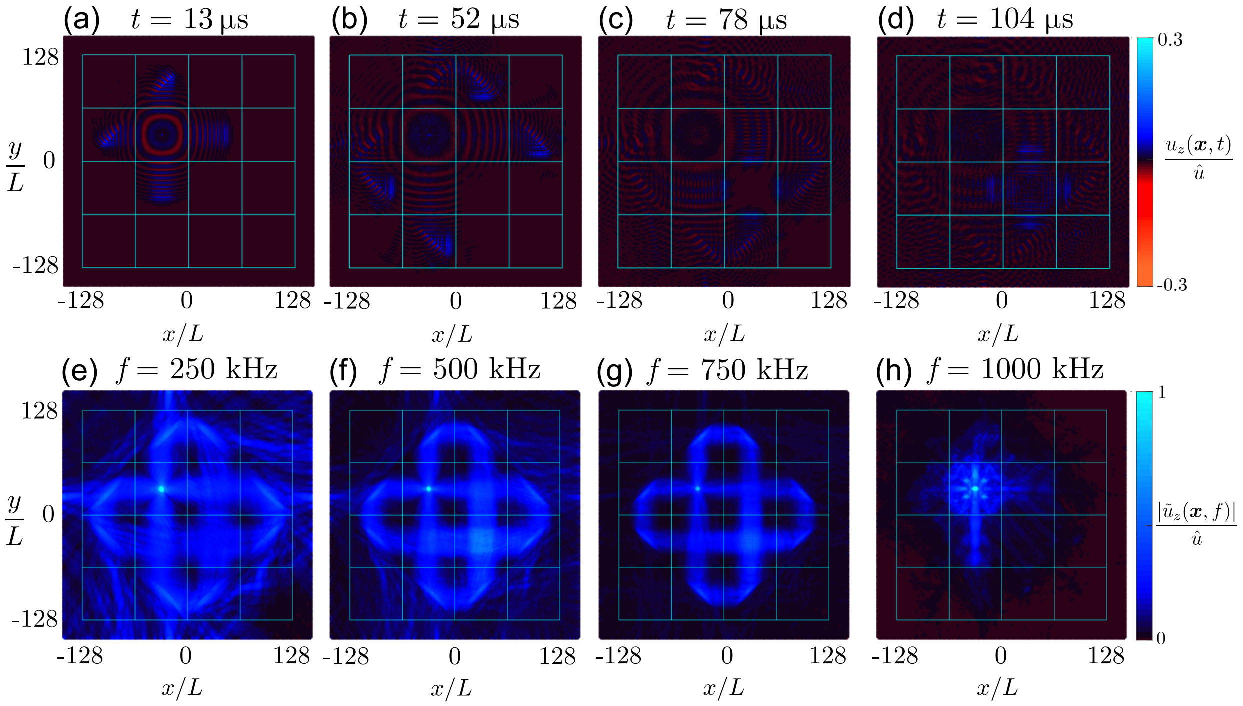


**Supplementary Figure 4: Broadband transient simulation results for the cross design.** Snapshots of the wave field in the transient dynamic finite element simulation of the cross design at (a) 13, (b) 52, (c) 78, and (d) 104 $\mu$s. The amplitude of the temporal FFT of the displacement signal at each spatial location is plotted at (e) 250, (f) 500, (g) 750 (the design frequency), and (h) 1000 kHz.

In the context of inverse problems, the availability of sensitivities via the adjoint state method from Eq. (S11) further benefits the efficiency of gradient-based optimization (e.g., compared to finite difference derivative computation). As an example, evaluation of the cost function and its gradient with respect to the 3844 design variables for the Tile 1 solution, when ray tracing is parallelized on 14 cores, takes 8.8 seconds. (We note that this computation time for the cost function and its gradient varies throughout the optimization.)

# Supplementary Note 3 Microfabrication

Samples were fabricated using commercially-procured 100 mm diameter Silicon-On-Insulator (SOI) wafers with three layers: a single-crystal silicon device layer (thickness $10 \pm 0.5$ µm), a buried oxide (BOX) layer (thickness $2.4 \pm 0.12$ µm), and a single-crystal silicon handle layer (thickness $400 \pm 10$ µm). The fabrication protocol involved etching windows in the handle layer, the designed architecture in the device layer, and release of the architected device layer by etching away the intermediate BOX layer. The following sequence of steps was followed during fabrication.

- **Back window etching**: The first sequence of steps was developed to etch windows into the device layer for pump excitation of the acoustic wave (see Methods section in the main paper). Before this sequence, a thin layer ($\sim 3$ µm) of sacrificial positive photoresist (AZ4533) was deposited and baked (120°C for 2 minutes) on the device layer to protect this surface from damage or contamination. A photomask was custom designed and written by a direct laser writer (DLW), commonly used for optical lithography, at the BRNC Cleanroom facility in IBM Zurich. The handle layer was spin-coated with positive photoresist AZ4562, exposed using the photomask, and developed by a standard photolithography protocol (developer: dilute KOH solution (AZ400K, 1:3 ratio) for $\approx 40$ seconds), followed by Deep Reactive Ion Etching (DRIE) for 60-90 minutes.

- **Device etching**: The next sequence of steps involved etching the architecture in the device layer, again using a high-resolution custom-developed photomask. A home-built Python code generates the input files for photomask writing, using the computational design output files. This infrastructure allows for the seamless handover between computational design and fabrication, which may also be automated. The same standard photolithography techniques were used as in the previous step, albeit using a different photoresist (AZ4533), hence a shorter time for development (25 seconds). This was followed by DRIE for $\sim 15$ minutes.
- **Photoresist stripping**: Photoresist layers were carefully stripped by first rinsing with acetone and isopropyl alcohol, followed by immersion in dimethyl sulfoxide (DMSO) at 120°C for $\sim 10$ minutes.
- **Release and post-fabrication treatment**: The intermediate BOX layer was removed using dry HF etching to generate free-standing architected films of 10 µm thickness. As a final step, $20 - 50$ nm thin aluminum films were deposited on each side of the sample to minimize the penetration of lasers during acoustic pump-probe measurements (see the Methods section of the main text for details).

# Supplementary Note 4 Data analysis

Standard Fourier analysis was performed on the raw time series data (averaged over 50 samples) at each measurement point. Due to the broadband excitation of the experiment relative to the computational design, each data set was filtered using a Butterworth bandpass filter [14] of order 15 — the choice of filter order was to minimize the loss of signal at the edges of the pass band. For the data presented in Fig. 4 of the main text, a pass band of width 1.6 MHz centered at 1.4 MHz was chosen.

Broadband excitation can provide further insight into the frequency-dependent wave-guiding response of the sample. To this end, data was further filtered using narrower pass bands with multiple center frequencies $\omega_c$ between 400 kHz and 1.5 MHz, and width $\Delta\omega = 200$ kHz. Results of this analysis and comparison with FE simulation data are discussed in Sec. Supplementary Note 5. FE simulation data were analyzed using the same parameters and numerical framework as the experimental data.

# Supplementary Note 5 Frequency-dependent wave guiding: experiments vs. simulations

Figs. 5, 6, and 7 show results from the frequency-dependent analysis described in the previous section along lines L1, L2, and L3, respectively, as defined in the main text. Each top row shows data from experiments, while the corresponding bottom row shows equivalent FE simulation data. Each column corresponds to a specific center frequency (a constant filter width of 200 kHz was maintained). In general, we observe excellent agreement between experimental and simulated data. Note that the computational inverse design was for a target frequency of 750 kHz.

Line scans L2 (Supplementary Fig. 6) do not show significant differences from the broadband data (Fig. 4 of the main manuscript) at the center frequencies $\omega_c = 0.5$ MHz and 0.7 MHz. At 0.9 MHz and 1 MHz, however, we notice that there is almost no visible signal beyond the excitation point. This is consistent with the observations from line scan L1 (Supplementary Fig. 5): wave modes at higher frequencies (close to 1 MHz) seem to be "trapped" within line L1 and the horizontal line connecting the centers of L1 and L2. Hence, high-frequency waves are trapped between two perpendicular lines intersecting at the excitation point, with waves being attenuated before the reflection points expected from the figure-eight wave guiding response.

This observation is supported by data from line scan L3 (Supplementary Fig. 7), showing that this wave has attenuated significantly by the time it reaches the center of line L3. The latter is apparent from the

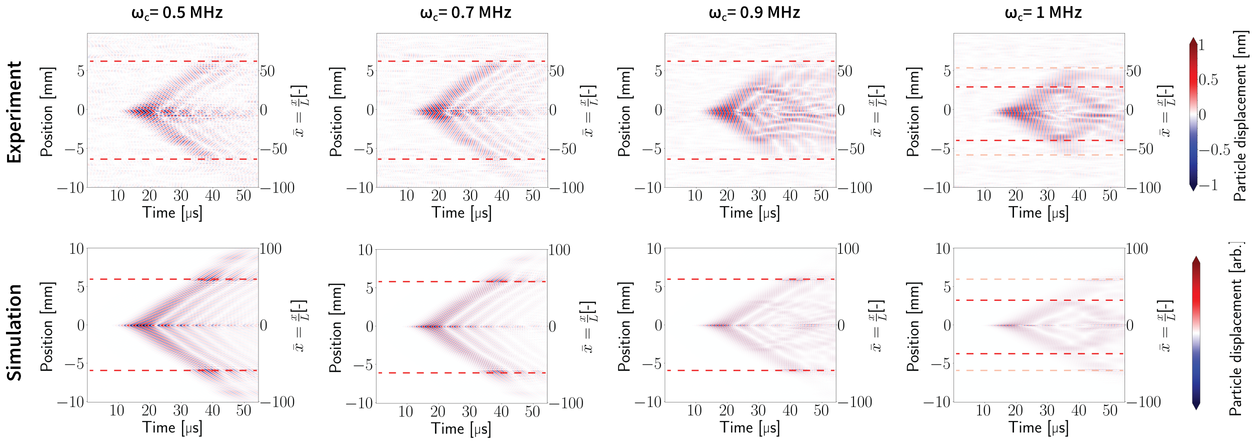


**Supplementary Figure 5: Experimental results for line scan L1.** Bandpass-filtered results from line scan L1 (filter width $\Delta\omega = 200$ kHz): comparison between experiment and simulation shows excellent agreement as well as wave guidance over a broad frequency range. The red dashed lines indicate the positions beyond which no wave propagation is observed. Above 0.8 MHz, back-reflection effects are noticeable in both experiments and simulations, which emerge within the figure-8 and shall not be confused with reflections from the outer boundaries.

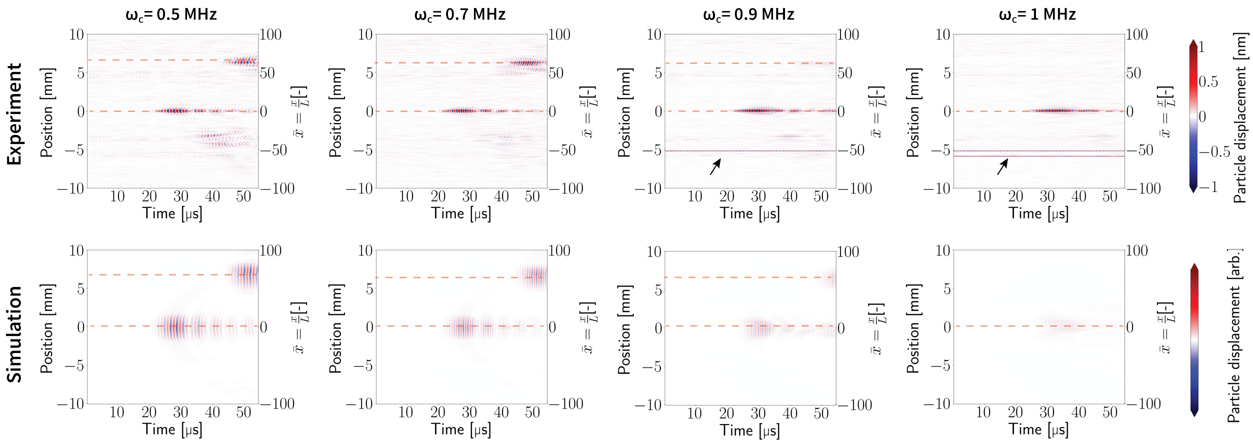


**Supplementary Figure 6: Experimental results for line scan L2.** Bandpass-filtered results from line scan L2 (filter width $\Delta\omega = 200$ kHz): comparison between experiments and simulations. The red dashed lines indicate the positions beyond which no wave propagation is observed. No significant signal is observed beyond the excitation point at center frequencies $\omega_c > 0.8$ MHz. Noisy data at two measurement positions, to be discarded, are marked by black arrows.

reduction in signal amplitude at center frequencies of 0.9 and 1 MHz in Supplementary Fig. 7. While this could in principle be an effect of other dissipation mechanisms (e.g., air damping), these effects were much less pronounced at lower frequencies, which hints at a structural, dispersive origin of the observed high-frequency wave attenuation through our graded architecture.

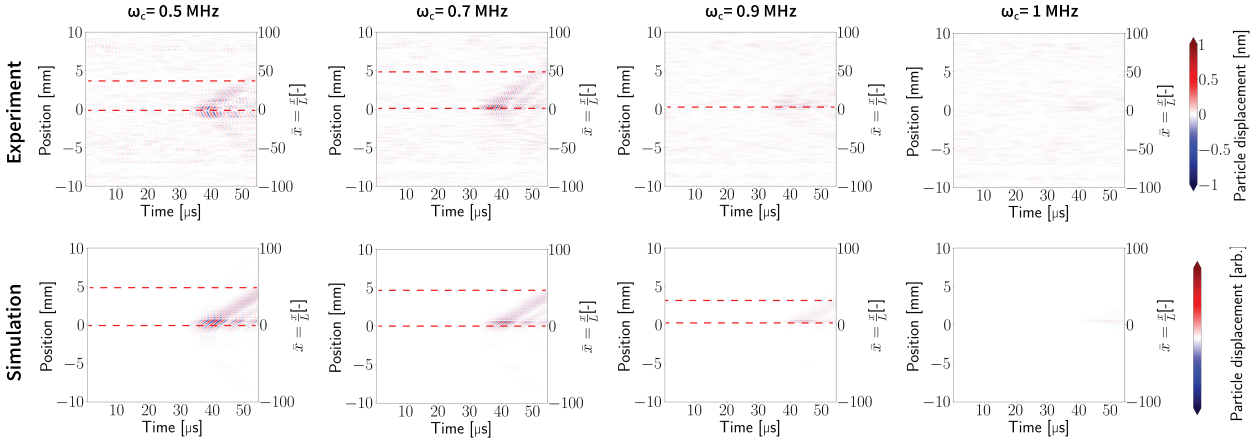


**Supplementary Figure 7: Experimental results for line scan L3.** Bandpass-filtered results from line scan L3 (filter width $\Delta\omega = 200$ kHz): comparison between experiments and simulations. The red dashed lines indicate the positions beyond which no wave propagation is observed. No significant signal is observed beyond the excitation point at center frequencies beyond $\omega_c > 0.8$ MHz. This confirms wave trapping along the horizontal $x$-axis at those frequencies.

# Supplementary References

[1] Bastian Telgen, Vignesh Kannan, Jean-Charles Bail, Charles Dorn, Hannah Niese, and Dennis M Kochmann. Rainbow trapping of out-of-plane mechanical waves in spatially variant beam lattices. *Journal of the Mechanics and Physics of Solids*, 191:105762, 2024.

[2] Charles Dorn and Dennis M Kochmann. Inverse design of graded phononic materials via ray tracing. *Journal of Applied Physics*, 134(19), 2023.

[3] Charles Dorn and Dennis M Kochmann. Conformally graded metamaterials for elastic wave guidance. *Extreme Mechanics Letters*, 65:102091, 2023.

[4] Charles Dorn and Dennis M Kochmann. Ray theory for elastic wave propagation in graded metamaterials. *Journal of the Mechanics and Physics of Solids*, 168:105049, 2022.

[5] Ari Ben-Menahem and Wafik B Beydoun. Range of validity of seismic ray and beam methods in general inhomogeneous media-i. general theory. *Geophysical Journal International*, 82(2):207–234, 1985.

[6] Mikhail M Popov and Christian Camerlynck. Second term of the ray series and validity of the ray theory. *Journal of Geophysical Research: Solid Earth*, 101(B1):817–826, 1996.

[7] Arjun Teh, Matthew O'Toole, and Ioannis Gkioulekas. Adjoint nonlinear ray tracing. *ACM Transactions on Graphics*, 41(4):1–13, 2022.

[8] Dong C Liu and Jorge Nocedal. On the limited memory BFGS method for large scale optimization. *Mathematical Programming*, 45(1):503–528, 1989.

[9] Steven G. Johnson. The NLopt nonlinear-optimization package, 2007. https://github.com/stevengj/nlopt.

[10] Mechanics and Materials Lab. ae108, 2020. https://doi.org/10.5905/ethz-1007-257.

[11] Matthew A Hopcroft, William D Nix, and Thomas W Kenny. What is the Young's modulus of silicon? *Journal of Microelectromechanical Systems*, 19(2):229–238, 2010.

[12] A Srikantha Phani, J Woodhouse, and NA Fleck. Wave propagation in two-dimensional periodic lattices. *The Journal of the Acoustical Society of America*, 119(4):1995–2005, 2006.

[13] Alex J Zelhofer and Dennis M Kochmann. On acoustic wave beaming in two-dimensional structural lattices. *International Journal of Solids and Structures*, 115:248–269, 2017.

[14] butter &x2014; SciPy v1.15.2 Manual — docs.scipy.org. https://docs.scipy.org/doc/scipy/reference/generated/scipy.signal.butter.html. [Accessed 17-04-2025].
