# Supplementary material for: Graded phononic metamaterials based on scalable microfabrication and design
Source: Nat Commun. 2026 Feb 25;17:3192. doi: 10.1038/s41467-026-69888-x (PMC13057018; doi:10.1038/s41467-026-69888-x)
Supplement: Supplementary file 2 — Description of Additional Supplementary Files [file 41467_2026_69888_MOESM2_ESM.pdf]

Description of Additional Supplementary Files

File Name: Supplementary Movie 1
Description: **Transient simulation of the figure-eight waveguide design.** A transient finite simulation performed on the fully-resolved microstructure of the figure-eight design (main text Fig. 2c) is visualized. Beam elements are used, and a harmonic out-of-plane displacement excitation is applied to the center point of the design with frequency 750 kHz and amplitude $\hat{u}$. The normalized out-of-plane displacement $u_z/\hat{u}$ is animated through time as the wave emanates from the point excitation and is guided along the figure-eight trajectory. The boundaries of the 9 tiles that make up this design are outlined.

File Name: Supplementary Movie 2
Description: **Transient simulation of the cross waveguide design.** A transient finite simulation performed on the fully-resolved microstructure of the cross design of main text (main text Fig. 2f) is visualized. Beam elements are used, and a harmonic out-of-plane displacement excitation is applied to the center point of the design with frequency 750 kHz and amplitude $\hat{u}$. The normalized out-of-plane displacement $u_z/\hat{u}$ is animated through time as the wave emanates from the point excitation and is guided along the cross-shaped trajectory. The boundaries of the 16 tiles that make up this design are outlined.
